# Modern Sociology

**Published:** 1898-10-29

**Authors:** 


### Modern Sociology.

### JUVENILE CRIMINALS.

The *Times* of September 24th contains a very suggestive letter by the Secretary of the Howard Association on the subject of "juvenile offenders and short sentences." The two themes, though closely allied, are really quite distinct, as the writer proves, when, after speaking of the different causes of juvenile crimes, he proceeds to deal with the result of short sentences on adult criminals, as shown in the statistics most usefully collected by the Hon. Captain Anson, Chief Constable of Stafford. We consider that both matters can be most efficiently treated separately, and therefore we will turn first to the vexed question of a remedy for juvenile crime. The primary cause of it is attributed by many who have studied the subject to the widespread disregard of the provisions of the Education Act. The Commissioners find "from figures recently furnished that at the present time nearly three-quarters of a million of children (about one-eighth of the child population of the country), whose names ought to be on the books of some elementary school, do not appear there at all." This is certainly a serious fact, and there are various causes to which it may be attributed. First, we must take into account the vast proportion of children of the lowest class, who escape the provisions of the Education Act altogether, because they inhabit wandering vans and canal boats, besides the great number who belong to the army of tramps, and are never long enough in one place to come under any authoritative supervision at all. Secondly, there is the very great prevalence of truancy on the part of children whose names are on the school books, but who, being left to their own devices, prefer the street gutters as a playground to those seats of learning. For this last neglect the parents are undoubtedly responsible, but the universal abnegation of that responsibility is a fact patent to all. How often is the phenomenon witnessed in the police-courts of a stalwart English father mournfully assuring the magistrates that a diminutive rascal of eight or nine years old is beyond his control. Both these causes of the abandonment of innumerable children to a condition utterly devoid of training or education might be successfully met if the plan effectively carried out in France, under the name of *L'assistance paternelle du Gouvernment*, were to be adopted in England. Under that wise control, the existence of children in caravans and coal barges could not be ignored, as it often is in this country; nor could helplessly or criminally neglectful parents prevent their children from being paternally cared for by the Legislature. Nor is it only in the prevention of childish crimes by judicious training that France manages such matters better than we do. Their manner of dealing with juvenile iniquity when finally developed in action is much more efficacious than ours. Many of our magistrates who have to deal with juvenile crime are strongly prejudiced against reformatories, and they have a yet more emphatic dislike to the idea of sending young boys or girls to prison. And what is the result? That their mode of dealing with very serious offences is almost ludicrous in its inefficiency. We may take as an instance a case which occurred the other day; a vicious little boy, nine years of age, deliberately placed a number of stones on the railway line with the settled purpose of causing an accident to the express. A serious catastrophe was only averted by the promptitude of a lady passing that way, who removed them at once. The small villain was then sitting on a gate near, waiting to see the havoc he had prepared. When she asked him who put the stones on the rails, he said he did not know, but happening to look back, when she had passed on, she saw him rush down to the line and endeavour to replace all the stones as before. Happily he had not time to fully accomplish his task, so that the engine of the express was able to crash over part of the stones, with only the result of heavy jolting and alarm to the passengers—a very slight difference in the position of the obstruction must have resulted in a terrible loss of life. Now when this young would-be murderer was brought before the magistrate, accompanied by the usual feebly apologetic parent, the judicial arbiter of his fate stated that he disapproved of reformatories, and awarded the culprit —who openly avowed that it was his habitual practice to attack trains—the punishment of six strokes with a birch rod, after which he was to resume his career of liberty and incipient vice. The birching, however sharp during the few minutes it lasted, was not likely to produce a life-long impression on a mind of nine years maturity, and certainly could not be considered an adequate substitute for four or five years' training in a reformatory. We do not deny that these institutions might be improved; but the prejudice which is setting in against them is probably to some extent due to the feeling excited on the subject of prison reform since the question was brought up in the House of Commons some months ago. Whatever be the cause of the distrust of reformatories, it admits, however, of a remedy. We have already in these columns, some time since, drawn attention to the admirable system for training children socially liable to evil influences, in classified Homes, to which in France they are assigned according to their propensities and the conditions of their existence. These Homes are conducted entirely by women, *religieuses* for the most part, but they have proved to undeniable demonstration that up to a certain age feminine influence is incomparably the most successful in the training of boys. We do not ourselves entertain the objection to the existing reformatories which obtains so largely among our legislators, but we do emphatically desire that the employment of female managers, which is shown to be so successful on the other side of the Channel, might be definitely adopted in our own land. We have exhausted our space in dealing with the first part of our subject, and therefore must leave the consideration of short sentences to our next issue.

